# JQ1 Inhibits the Hyperproliferation and Aggressiveness of Fibroblast‐Like Synoviocytes in Rheumatoid Arthritis via Regulating IL7R

**DOI:** 10.1002/iid3.70540

**Published:** 2026-10-28

**Authors:** Lin Hong, Tingting Chen, Yuchen Jiang, Xiaohua Zhang

**Affiliations:** ^1^ Department of Pharmacology Wenzhou Hospital of Integrated Traditional Chinese and Western Medicine Wenzhou China; ^2^ Department of Pharmacy, the Fourth Affiliated Hospital of School of Medicine, and International School of Medicine, International Institutes of Medicine Zhejiang University Jinhua China

**Keywords:** fibroblast‐like synoviocytes, IL7R, JQ1, rheumatoid arthritis

## Abstract

**Background:**

Rheumatoid arthritis (RA) is a chronic autoimmune bone disease. BRD4 is implicated in the pathogenesis of bone disorders. This study aimed to investigate the effects of a BRD4 inhibitor JQ1 on RA.

**Methods:**

Fibroblast‐like synoviocytes (FLS) were exposed to TNF‐α to establish an in vitro RA model. mRNA levels were detected using RT‐qPCR. Protein expression was assessed by Western blot. The behaviors of FLS were analyzed using CCK‐8, EdU, wound scratch, and transwell assays.

**Results:**

We found that LPS exposure promoted an inflammatory response in FLS, as well as mediated cell proliferation and migration. Interestingly, JQ1 inhibited the inflammatory response and the morbidity of FLS. Mechanistically, targeting BRD4 by JQ1 inhibited acetylation modification for H3K27 on IL7R promoter, downregulating the expression of IL7R. However, IL7R overexpression reversed the effects of JQ1 and promoted the inflammatory response and the morbidity of FLS.

**Conclusion:**

JQ1 inhibits the inflammatory response, proliferation, and migration of RA FLS via downregulating IL7R. Therefore, JQ1 exerts protective effects on RA.

## 1 Introduction

Rheumatoid arthritis (RA) affects approximately 1% of the global population, representing a substantial burden on healthcare systems [1]. RA is characterized by symmetric polyarthritis, systemic inflammation, and the progressive erosion of articular cartilage and bone [2]. Despite significant advances in therapeutic strategies, including the widespread use of disease‐modifying antirheumatic drugs and biologic agents, a considerable proportion of patients fail to achieve sustained remission or experience adverse effects that limit treatment efficacy [3, 4, 5]. The pathological hallmark of RA is the formation of invasive pannus tissue, which mediates joint destruction through the coordinated action of multiple cell types, including macrophages, T lymphocytes, B cells, and fibroblast‐like synoviocytes (FLS) [6, 7, 8]. Among these cellular constituents, FLS have garnered increasing attention due to their unique contribution to disease perpetuation and tissue damage [9].

Interleukin‐7 is a pleiotropic cytokine traditionally recognized for its essential role in lymphocyte development and homeostasis [10]. However, accumulating evidence implicates IL‐7 and its receptor complex (IL7Rα/CD127 and common γ‐chain/CD132) in RA pathogenesis [11, 12, 13]. Elevated IL‐7 levels have been detected in synovial fluid and serum of RA patients [14]. In the context of RA, IL7R enhances FLS proliferation and survival through activation of anti‐apoptotic pathways [15]; IL7R stimulates the production of pro‐inflammatory cytokines by FLS [16]; IL7R promotes T cell survival and activation within the synovial microenvironment [17]. The IL‐7/IL7R axis thus represents an attractive therapeutic target for modulating both synovial inflammation and structural damage in RA.

Bromodomain‐containing proteins, particularly the BET family (BRD2, BRD3, BRD4, and BRDT), function as critical epigenetic readers that recognize acetylated lysine residues on histone tails and facilitate the assembly of transcriptional complexes at specific genomic loci [18]. JQ1 is a selective and cell‐permeable inhibitor of BET bromodomains [19]. By competitively displacing BET proteins from chromatin, JQ1 suppresses the transcription of target genes [20]. Recently, JQ1 has shown promise in inflammatory and autoimmune conditions, including amyotrophic lateral sclerosis, inflammatory bowel disease, and RA [21, 22, 23].

Given the established roles of BET proteins in regulating inflammatory gene expression and the emerging importance of IL7R signaling in RA pathogenesis, we hypothesized that JQ1 might exert therapeutic effects in RA by modulating IL7R expression and function in FLS.

## 2 Materials and Methods

### 2.1 Cell Culture

Primary FLS were isolated from synovial tissues obtained from mice. Cells were maintained in DMEM (Gibco, USA) supplemented with 10% FBS, 100 U/mL penicillin/streptomycin at 37°C in 5% CO_2_. Cells were treated with TNF‐α (50 ng/ml) (Sigma‐Aldrich, Germany) for 24 h or BRD4 inhibitor JQ1 (1 μM) (Sigma‐Aldrich, Germany). Cells at 6 passages were used for further experiments.

### 2.2 Transfection

RA‐FLS were transfected with IL7R overexpression plasmids/BRD4 shRNAs or the control (GenePharm, China) using Lipofectamine 3000 (Invitrogen, USA) for 48 h.

### 2.3 qRT‐PCR

Total RNA was isolated using TRIzol reagent (Invitrogen). RNA was reverse‐transcribed using a cDNA synthesis Kit (Applied Biosystems, USA). Quantitative PCR was performed using SYBR Green Master Mix (Thermo Fisher Scientific, USA) on a QuantStudio 7 Flex Real‐Time PCR System (Thermo Fisher Scientific, USA). Relative expression was calculated using the 2^‐ΔΔCt^ method. GAPDH served as a loading control.

### 2.4 Determination of Oxidative Stress

The content of ROS, MDA, SOD, and T‐AOC was analyzed using the corresponding kits (Abcam, UK).

### 2.5 ELISA

Cytokine release was analyzed using the ELISA kits (Abcam, UK).

### 2.6 Western Blot

Cells were lysed in RIPA buffer. Protein concentrations were determined using the BCA protein assay. Protein was separated by 10% SDS‐PAGE and transferred to PVDF membranes. Afterwards, membranes were sealed with 5% skimmed milk, incubated with primary antibodies, followed by secondary antibodies. The blots were imaged using ECL reagents (Abcam, UK).

### 2.7 ChIP Assay

Cells were fixed with 1% formaldehyde. Chromatin was lyzed with the lysis buffers, and DNA was digested to ∼500 bp chromatin fragments. After centrifugation, the lysates were diluted. The supernatants were incubated with A–agarose beads conjugated with antibody against anti‐IgG, anti‐H3K27ac, or anti‐BRD4 (Abcam, UK). Then bead‐bound DNA was treated with ribonuclease A and protein K. The immunoprecipitated complexes were purified and subjected to PCR.

### 2.8 Cell Proliferation Assays

FLS were seeded in 96‐well plates and cultured for 72 h. CCK‐8 solution (0.5 mg/mL) was added to each well, and cells were incubated for 4 h. Finally, the absorbance was measured at 450 nm using a microplate reader.

### 2.9 Migration and Invasion Assays

Cell migration was evaluated using wound healing (scratch) assays and Transwell migration assays. For wound healing assays, confluent FLS monolayers were wounded using a sterile 200 μL pipette tip, washed with phosphate‐buffered saline (PBS) to remove detached cells, and cultured in serum‐free medium containing JQ1 or vehicle. Wound closure was monitored by phase‐contrast microscopy at 0 and 24 h. The percentage of wound closure was calculated using ImageJ software.

Transwell invasion assays were performed using 8.0 μm pore size polycarbonate membranes. FLS (5 × 10^4^ cells) suspended in serum‐free medium were seeded in the upper chamber pre‐coated with Matrigel (1 mg/mL), while the lower chamber contained medium with 10% FBS as a chemoattractant. After 24 h of incubation with JQ1 treatment, non‐migrated cells were removed from the upper surface, and migrated cells on the lower surface were fixed with 4% paraformaldehyde, stained with crystal violet, and counted under a light microscope.

### 2.10 Animal Models

Male DBA/1 J mice (8 weeks) were provided by Beijing Vital River Laboratory (Beijing, China). Mice were housed in specific pathogen‐free facilities. Mice were randomized into three groups: (1) Normal control (DBA1) group (*n* = 5), (2) CIA group (*n* = 5), and (3) CIA + JQ1 group (*n* = 5). Mice in CIA group were immunized with bovine type II collagen emulsified in complete Freund's adjuvant (CFA) on day 0, followed by a booster injection on day 21. Mice in CIA + JQ1 group were immunized with bovine type II collagen emulsified in complete CFA and intraperitoneally injected with JQ1 (10 mg/kg) daily.

### 2.11 Histological Analysis

Joint tissues were fixed in 4% paraformaldehyde, decalcified in 10% EDTA, and embedded in paraffin. The sections were stained with hematoxylin and eosin (H&E). The image was captured using a microscope (Leica, Germany).

### 2.12 Statistical Analysis

Each independent experiment was performed in triplicate. All data are presented as mean ± SD. Two‐group comparisons were analyzed using Student's *t*‐test. Multi‐group comparisons were analyzed using ANOVA followed by Tukey's post hoc test. A value of *p* < 0.05 were considered statistically significant. All statistical analyses were performed on GraphPad Prism 8.0.

## 3 Results

### 3.1 JQ1 Suppresses the Hyperproliferation and Aggressiveness of RA‐FLS

To evaluate the impact of BET inhibition on RA‐FLS proliferation, we first examined the effects of JQ1 on cell growth using multiple complementary assays. JQ1 treatment significantly reversed the effects of TNF‐α exposure and inhibited the cell viability of RA‐FLS (Figure 1A). The aggressive migratory behavior of RA‐FLS is a critical determinant of pannus invasion and joint destruction. JQ1 treatment significantly inhibited the migration of RA‐FLS compared with TNF‐α group (Figure 1B‐C). Moreover, JQ1 treatment significantly inhibited reduced the number of invaded RA‐FLS (Figure 1D‐E). These results suggested that JQ1 aggressive phenotype of RA‐FLS.

**Figure 1:**
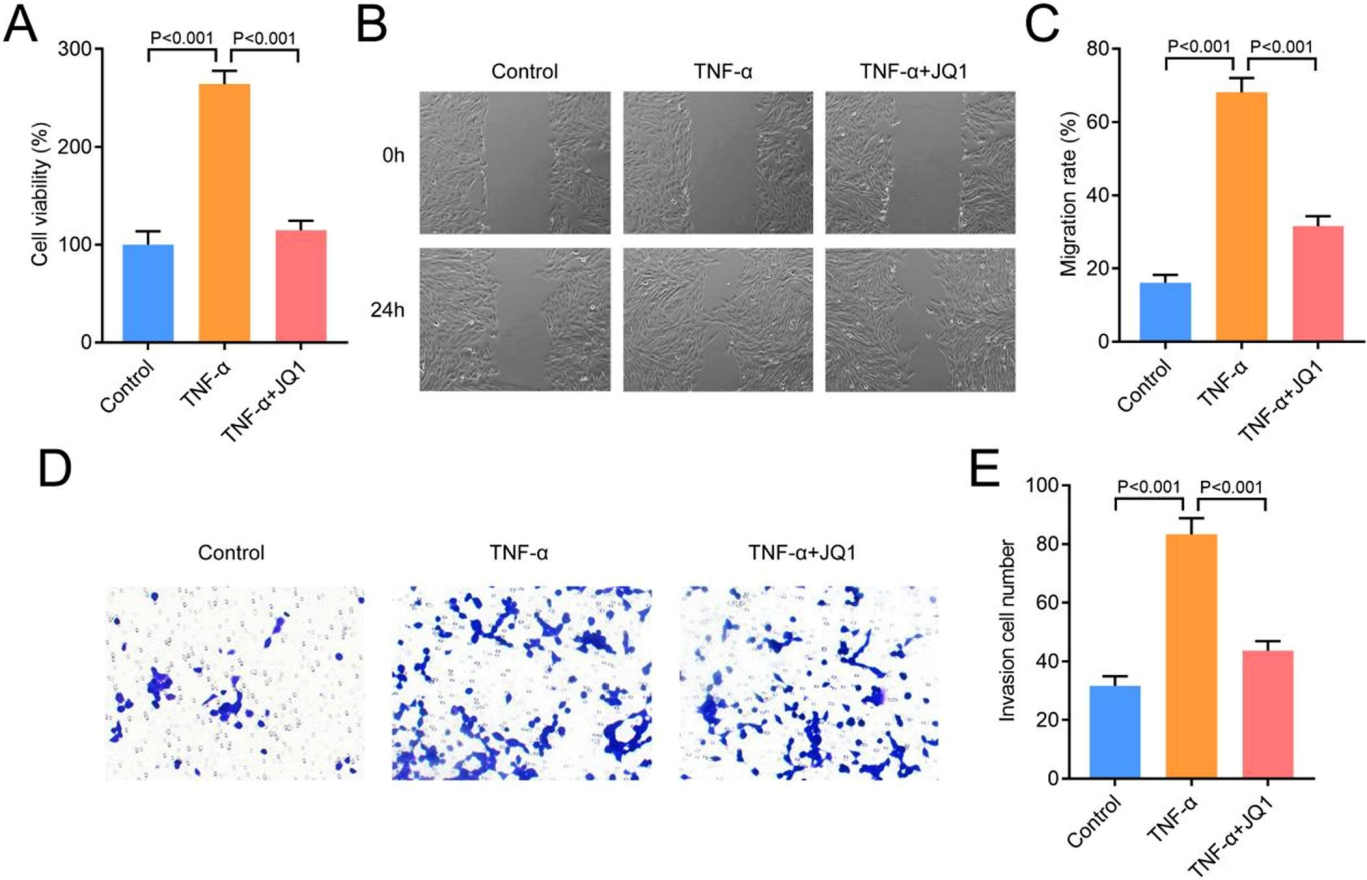
*JQ1 Suppresses the Hyperproliferation and Aggressiveness of RA‐FLS*. (A) Cell viability was analyzed using CCK‐8 assay. (B, C) Cell migration was detected using a wound scratch assay. (D, E) Cell migration was detected using a transwell assay. *p* < 0.001.

### 3.2 JQ1 Attenuates the Oxidative Stress and Inflammatory Response in RA‐FLS

Oxidative stress is involved in the progression of RA pathogenesis [24]. As shown in Figure 2A, the content of ROS was significantly increased by TNF‐α exposure, which was reversed by JQ1 treatment. JQ1 treatment significantly inhibited TNF‐α exposure‐mediated lipid peroxidation (Figure 2B). Moreover, JQ1 treatment significantly increased the levels of antioxidant SOD and T‐AOC compared with TNF‐α group (Figure 2B). The invasiveness and pro‐inflammatory properties of RA‐FLS constitute primary factors driving synovial inflammation and articular cartilage damage during the course of RA progression [25]. We found that JQ1 treatment significantly reduced the release of pro‐inflammatory IL‐6 and IL‐1β (Figure 2C), whereas increasing the release of anti‐inflammatory IL‐10.

**Figure 2:**
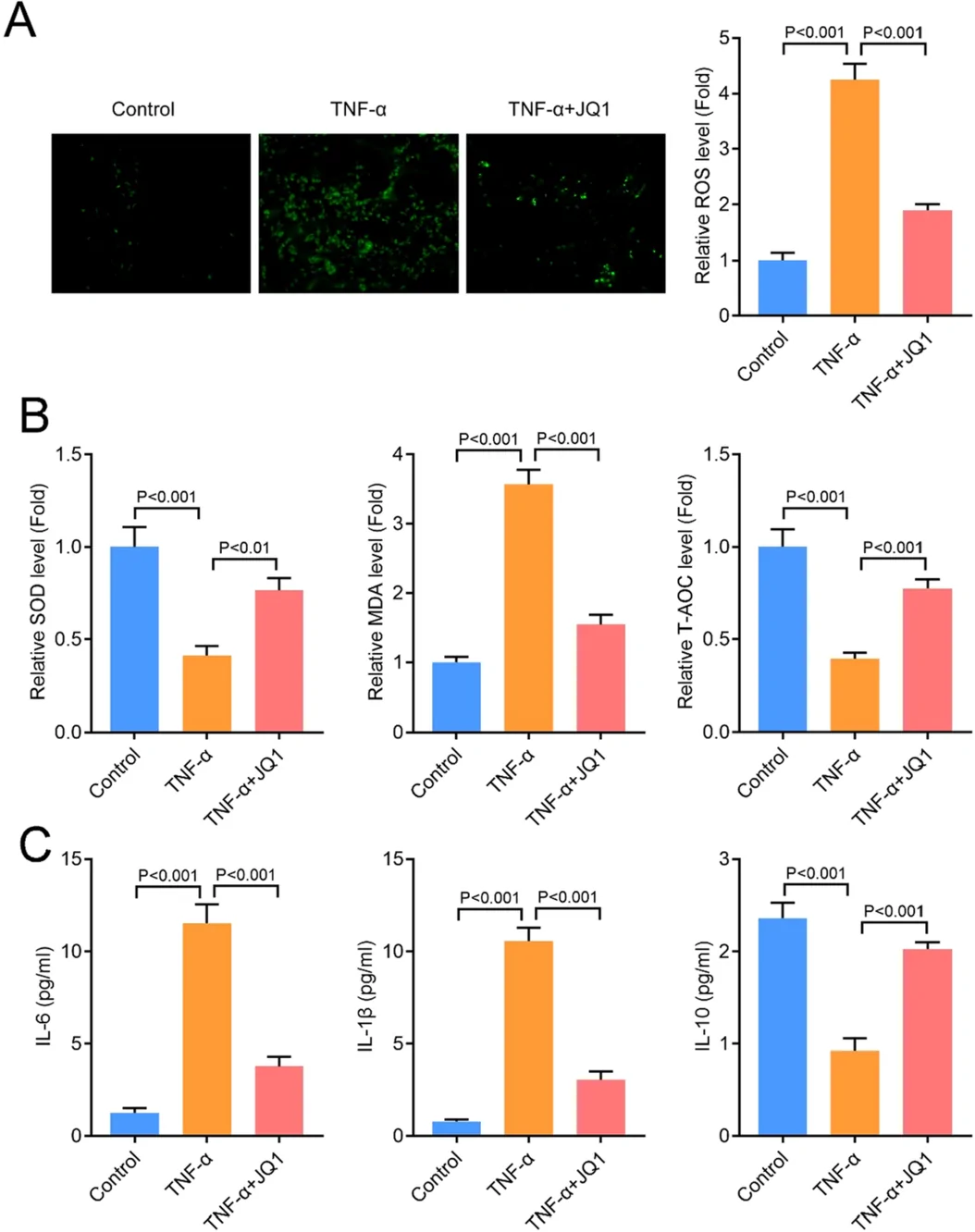
*JQ1 Attenuates the Oxidative Stress and Inflammatory Response in RA‐FLS*. (A) The fluorescence images (left) and quantitative analysis of ROS (right). (B) The levels of SOD, MDA, and T‐AOC. (C) Cytokine release was analyzed using ELISA. *p* < 0.001.

### 3.3 JQ1 Downregulates IL7R Expression in RA‐FLS

JQ1 is an inhibitor of BRD4. To explore the molecular mechanisms underlying the effects of JQ1 on FLS behavior, we examined the expression of IL7R, given its established role in promoting FLS activation and survival. We hypothesized that BRD4‐IL7R axis may contribute to RA. We found that BRD4 and H3K27ac were selectively recruited in the promoter of IL7R (Figure 3A‐B). TNF‐α exposure‐mediated upregulation of IL7R was significantly reversed by BRD4 shRNAs (Figure 3C). Moreover, JQ1 treatment significantly inhibited the recruitment of BRD4 and H3K27ac in the promoter of IL7R (Figure 3D‐E). Additionally, JQ1 treatment resulted in a substantial reduction in IL7R mRNA levels (Figure 3A). Western blot analysis confirmed that JQ1 decreased IL7R protein expression in a dose‐ and time‐dependent manner (Figure 3B‐C).

**Figure 3:**
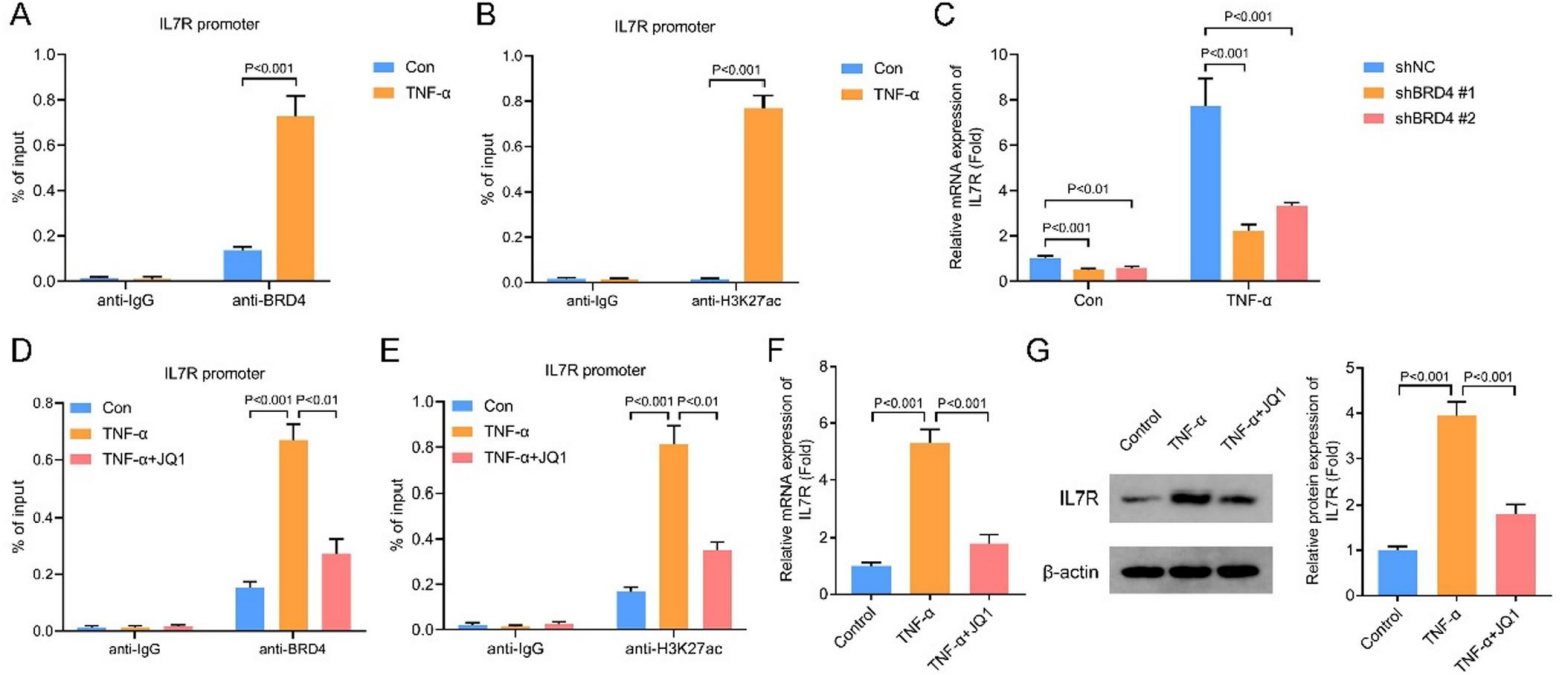
*JQ1 Downregulates IL7R Expression in RA‐FLS*. The enrichment of BRD4 (A) and H3K27ac (B) in the promoter of IL7R was analyzed using ChIP assay. (C) IL7R mRNA levels were analyzed using qRT‐PCR. The enrichment of BRD4 (D) and H3K27ac (E) in the promoter of IL7R was analyzed using ChIP assay. (F) IL7R mRNA levels were analyzed using qRT‐PCR. (G) IL7R protein expression was detected using Western blot. *p* < 0.001.

### 3.4 IL7R Alleviates the Effects of JQ1 on FLS Proliferation and Migration

To determine whether the suppression of IL7R contributes to the functional effects of JQ1 on FLS, we performed rescue experiments using IL7R overexpression. IL7R mRNA levels were significantly increased by its overexpression plasmids (Figure 4A), suggesting successful transfection. IL7R overexpression significantly abrogated the effects of JQ1 and increased the cell viability of RA‐FLS (Figure 4B). Moreover, IL7R overexpression significantly promoted the migration and invasion of RA‐FLS compared with TNF‐α + JQ1 + Vector group (Figure 4C‐D). Moreover, IL7R overexpression significantly increased oxidative stress (Figure 4E‐F) and lipid peroxidation in RA‐FLS compared with TNF‐α + JQ1 + Vector group (Figure 4G), while reduced the content of SOD and T‐AOC. Additionally, IL7R overexpression significantly promoted inflammatory response in RA‐FLS (Figure 4H).

**Figure 4:**
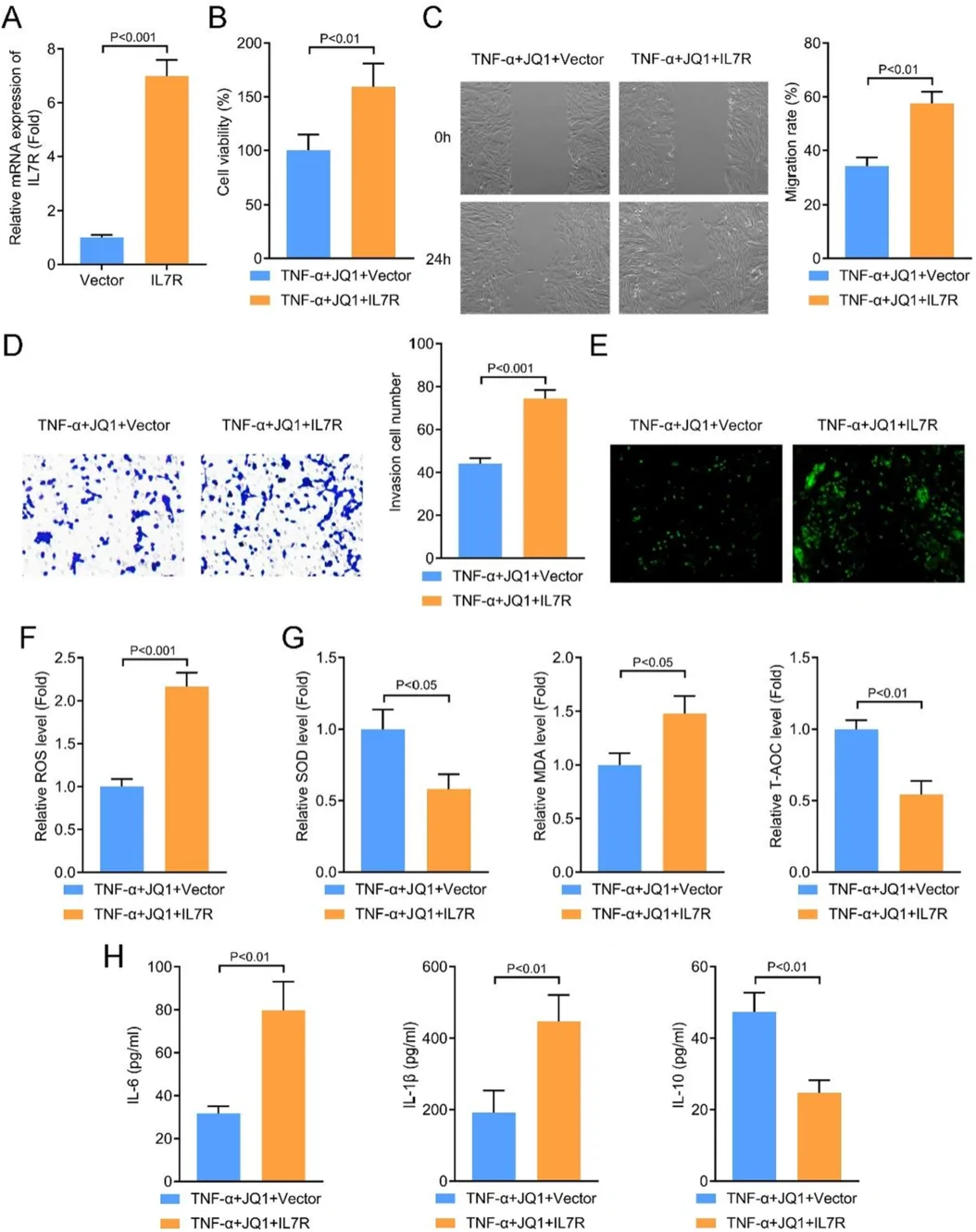
*IL7R Alleviates the Effects of JQ1 on FLS Proliferation and Migration*. (A) IL7R mRNA levels were analyzed using qRT‐PCR. (B) Cell viability was analyzed using CCK‐8 assay. (C) Cell migration was detected using wound scratch assay. (D) Cell migration was detected using transwell assay. The fluorescence images (E) and quantitative analysis of ROS (F). (G) The content of SOD, MDA, and T‐AOC. (H) Cytokine release was analyzed using ELISA. *p* < 0.05, *p* < 0.01, *p* < 0.001.

### 3.5 JQ1 Alleviates the Progression of RA In Vivo

To confirm the effects of JQ1 on RA, mice were treated with CIA plus JQ1. We found that JQ1significantly reduced the arthritis scores (Figure 5A). Moreover, CIA‐mediated an increase in paw thickness was significantly reversed by JQ1 (Figure 5B), indicating that JQ1 alleviated bone erosion induced by CIA. The accumulation of immune cells in the joints of CIA mice (Figure 5C), which was significantly antagonized by JQ1. Furthermore, JQ1 significantly inhibited the inflammatory response in the joints of CIA mice (Figure 5E‐F).

**Figure 5:**
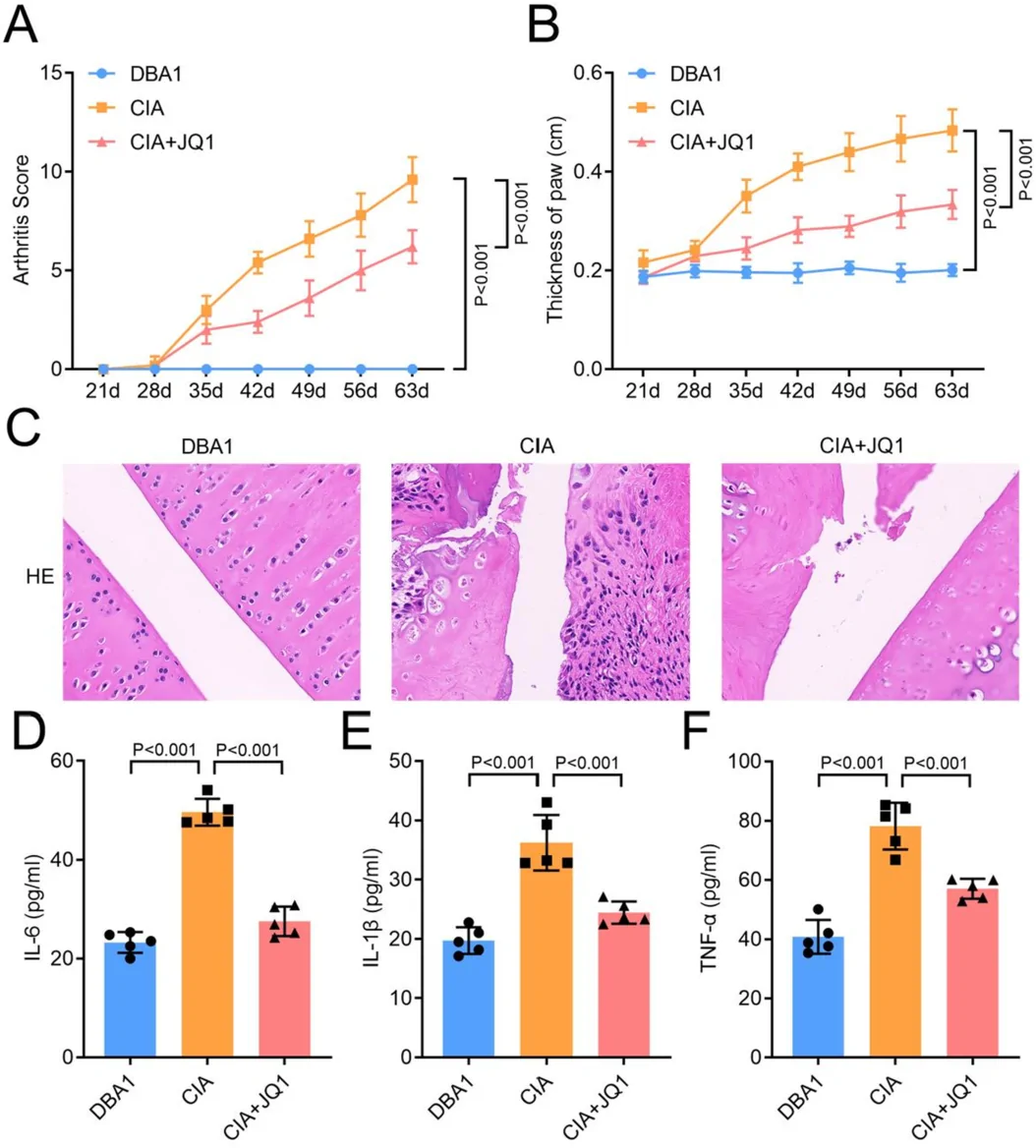
*JQ1 Alleviates the Progression of RA In Vivo*. (A) The arthritis scores of mice. (B) The paw thickness of mice. (C) Histological analysis was performed using HE staining. (D–F) Cytokine release was analyzed using ELISA. *p* < 0.001.

## 4 Discussion

The present study provides comprehensive evidence that the BET inhibitor JQ1 suppresses the pathogenic behavior of RA‐FLS through modulation of IL7R expression and signaling. In this study, JQ1 effectively inhibits RA‐FLS proliferation and induces cell cycle arrest without significant cytotoxicity. JQ1 attenuates FLS migration and invasion while reducing MMP production. JQ1 suppresses acetylation modification for H3K27 on IL7R promoter, downregulating IL7R expression. IL7R mediates a significant portion of the functional effects of JQ1 on FLS behavior. These findings establish a novel mechanistic link between BET inhibition and IL7R regulation, with important implications for RA pathogenesis and therapy.

The IL‐7/IL7R axis has emerged as a critical pathway in RA, contributing to multiple aspects of disease pathogenesis [26, 27]. Our findings extend previous observations by demonstrating that IL7R signaling promotes not only immune cell activation but also the aggressive behavior of FLS. The hyperproliferative and aggressive phenotype of RA‐FLS is partially maintained by autocrine or paracrine IL‐7 signaling, which creates a self‐perpetuating cycle of synovial activation. Interestingly, targeting BRD4 by its specific inhibitor JQ1 inhibits acetylation modification for H3K27 on IL7R promoter, downregulating IL7R expression.

The therapeutic potential of BET inhibitors in RA is supported by several lines of evidence from the present study. First, JQ1 effectively suppresses the cardinal features of RA‐FLS pathobiology, including hyperproliferation, migration, and invasive behavior. These cellular phenotypes are directly linked to pannus formation and joint destruction in vivo. Second, the effects of JQ1 on IL7R expression suggest that BET inhibition may modulate stromal cell compartments in RA. IL7R blockade has shown promise in preclinical models of autoimmunity [28]. By suppressing IL7R expression broadly, BET inhibitors may exert immunomodulatory effects in addition to their direct actions on FLS. Third, the cytostatic rather than cytotoxic nature of JQ1 effects on FLS suggests a favorable therapeutic index, as non‐dividing cells such as quiescent tissue‐resident cells would be spared. This property distinguishes BET inhibitors from conventional cytotoxic agents used in refractory RA. However, several considerations warrant caution in translating these findings to clinical practice. First‐generation BET inhibitors such as JQ1 have demonstrated limited pharmacokinetic properties and potential toxicities in clinical trials, including thrombocytopenia and gastrointestinal adverse effects [29]. Second, the broad spectrum of BET‐regulated genes raises concerns about unintended immunosuppression or metabolic effects. Third, the development of resistance mechanisms, including compensatory upregulation of alternative pathways, may limit long‐term efficacy. These challenges have motivated the development of next‐generation BET inhibitors with improved selectivity, duration of action, and safety profiles. Additionally, strategies such as PROTAC (proteolysis‐targeting chimera)‐mediated BET protein degradation or tissue‐specific delivery systems may enhance the therapeutic window [30].

Several limitations of the present study should be acknowledged. First, our experiments were conducted primarily using in vitro culture systems, which may not fully recapitulate the complex cellular interactions and microenvironmental cues present in the rheumatoid synovium. Second, the use of primary FLS from a limited number of donors may not capture the full heterogeneity of RA patient populations. Third, the pharmacokinetic and pharmacodynamic properties of JQ1 in vivo may differ from the concentrations used in cell culture studies.

## 5 Conclusion

This study demonstrates that the BET inhibitor JQ1 suppresses the hyperproliferation and migration of RA‐FLS through downregulation of IL7R expression and disruption of downstream signaling pathways. Our findings establish a novel mechanistic link between epigenetic regulation and cytokine receptor signaling in RA pathogenesis, with important implications for therapeutic development. The BRD4‐IL7R axis represents a promising target for intervention that may modulate both stromal cell activation and immune dysregulation in RA. Further investigation of BET inhibitors in preclinical disease models and clinical trials is warranted to evaluate their potential as disease‐modifying agents in RA.

## Author Contributions

**Lin Hong:** methodology, conceptualization, writing – original draft. **Tingting Chen:** methodology, writing – review and editing. **Yuchen Jiang:** methodology, writing – original draft. **Xiaohua Zhang:** methodology, writing – review and editing, writing – original draft.

## Funding

The authors have nothing to report.

## Ethics Statement

All animal experiments were approved by Laboratory Animal Welfare and Ethical of Guangzhou Miles Biosciences Application (MIS2026059) and strictly implemented in compliance with the NIH Guide for the Care and Use of Laboratory Animals. All procedures were performed in accordance with ARRIVE guidelines.

## Conflicts of Interest

The authors declare no conflicts of interest.

## Data Availability

The data that support the findings of this study are available from the corresponding author upon reasonable request.
